# Survivor Treatment Selection Bias in Evaluations of Virtual Transition of Care

**DOI:** 10.2196/97119

**Published:** 2026-09-10

**Authors:** Hongmei Yang, Christopher D Russell, Amy Arnder Greene, Angela D Humphrey

**Affiliations:** 1 Clinical Quality Analytics Advocate Health Charlotte, NC United States; 2 Pulmonary and Critical Care Advocate Health Charlotte, NC United States; 3 Quality Management Advocate Health Charlotte, NC United States

**Keywords:** transitions of care, telehealth, telemedicine, hospital medicine, quality and patient safety, health care use

In the study “Virtual Transition of Care Clinics and Associated Readmission Rates: 3-Year Retrospective Cohort Study” [1], the authors reported a significantly lower 30-day readmission rate among patients who participated in a virtual transition of care (VToC) follow-up compared with those who did not. Multivariable regression further suggested that the VToC follow-up was independently associated with reduced readmissions after adjusting for baseline risk and demographic factors.

However, this association may reflect an important source of selection bias—survivor treatment selection bias (also referred to as immortal time bias), in which the outcome influences exposure classification. This bias arises when exposure is defined during the outcome observation window rather than ensuring that exposure initiation precedes outcome assessment, allowing early events to determine exposure status and generate spurious protective associations [2-4]. In the study [1], both the VToC follow-up window and the 30-day readmission outcome period began at hospital discharge. Patients who were readmitted before their scheduled VToC follow-up typically had their appointments canceled or were documented as no-shows and were, therefore, classified as not having received a VToC follow-up by the study design. Consequently, early readmissions were disproportionately assigned to the non-VToC group, leading to an overestimation of readmission in that group.

Although the authors adjusted for baseline readmission risk using the LACE+ index and for demographic factors, bias arising from outcome-dependent exposure assignment cannot be remedied by conventional risk adjustment alone. Methodologic approaches such as intent-to-treat analysis based on referral status or time-varying Cox regression models that explicitly account for the timing of exposure initiation are recommended to address this form of bias.

We observed a similar pattern in an internal evaluation assessing whether completion of a virtual follow-up visit was associated with 30-day readmission among patients discharged after hospitalization for chronic obstructive pulmonary disease exacerbation. Analyses that classified patients based on visit completion suggested a substantial protective association. However, closer examination showed that many patients who did not complete follow-up had been readmitted before their scheduled visits, which were subsequently recorded as canceled or no-shows. When patients were instead analyzed according to referral status (intent-to-treat) or when exposure timing was modeled using a time-varying Cox regression framework, no association between virtual follow-up and 30-day readmission was observed.

The discrepancy between analyses that do and do not account for survivor treatment selection bias underscores the importance of temporal alignment between exposure and outcome assessment. In evaluations of virtual or postdischarge interventions, apparent benefits may arise primarily from systematic misclassification of patients who experience early readmission rather than from a true intervention effect. Explicitly addressing exposure timing is, therefore, essential, as this bias cannot be corrected by adjustment for baseline risk factors alone.

## Data Availability

Deidentified, aggregate data will be made available upon reasonable request to qualified investigators, subject to approval by the authors. Requests may be directed to the corresponding author via email.

## Abbreviations

VToC: virtual transition of care
